# Response to “Comment on ‘Associations of Physical Fitness During Childhood with Arterial Structure and Stiffness in Adolescence: An 8-Year Follow-up Study’”

**DOI:** 10.1186/s40798-026-01073-2

**Published:** 2026-07-31

**Authors:** Emilia Laitinen, Eero A. Haapala

**Affiliations:** 1 Faculty of Sport and Health Sciences, University of Jyväskylä, Jyväskylä, Finland; 2 Active Life Laboratory, South-Eastern Finland University of Applied Sciences, Mikkeli, Finland

Dear Editor,

We thank Sansum and colleagues [1] for their interest in our article [2] and for highlighting the importance of selecting maturation assessments that align with the characteristics of the sample and research question. We agree that maturation is an important consideration in all paediatric focussed research, and we welcome the opportunity to further discuss methodological decisions within the context of current evidence.

Hormonal changes during maturation may influence arterial properties altering arterial function and structures. However, only a few studies have directly connected hormonal maturation to arterial stiffness or structure in children and adolescents. In contrast, it is well-documented that arterial stiffness increases with increasing age, stature, and blood pressure [3]. Additionally, sex has been associated with carotid intima–media thickness (cIMT) in youth, with boys demonstrating greater thickness compared to girls [4]. Therefore, the extent to which hormonal maturation affects arterial stiffness and structure over somatic growth and aging is not well established. Because direct assessment of hormonal maturation is not often feasible, several previous studies have assessed maturation using secondary sex-characteristics and stages described by Tanner [5, 6]. Pubertal development using these stages can be assessed using several metrics, such as breast development in girls, testicular volume in boys, and pubic hair development in both sexes, and using a clinical or self-assessment. Nevertheless, often used pubic hair assessment has also limitations in pubertal development, as it also reflects adrenarche development [7, 8]. Unfortunately, several previous studies on the associations between maturation and arterial health using stages described by Tanner lack accurate reporting of pubertal status assessment. The rationale for choosing the method for pubertal status assessment and transparency in methodological description should be considered highly relevant, as the chosen approach influences how the findings are interpreted and may limit the ability to understand potential associations with arterial health.

The limited number of studies investigating pubertal stage and arterial measures suggest only small differences in arterial stiffness across different Tanner stages in adolescence [9]. Similarly, in our own study [2] we observed no statistically significant differences in arterial stiffness or structure across pubertal stages (*p* > 0.05). However, one study using self-reported Tanner staging suggested that more advanced pubertal development was associated with greater cIMT [10], while another suggested that sex differences in large artery stiffness wears off after puberty [11]. Overall, these mixed findings indicate that associations between pubertal stage and arterial measures may depend on how the maturation is characterised, highlighting the need for alternative approaches that more precisely capture maturational timing and tempo.

Assessment of pubertal development using the Tanner stages has some inherent limitations, as these stages apply only during the pubertal period and classify individuals categorically, potentially obscuring meaningful variation within each stage. As highlighted by Sansum and colleagues [1], anchoring maturation to peak height velocity (PHV) may provide a more dynamic indicator of growth and maturation. PHV also provides physiologically relevant information related to arterial stiffness and structure, as several hormonal and somatic changes also affecting arterial health occur around this period. Optimally, PHV should be assessed using the SuperImposition by Translation And Rotation (SITAR) model based on repeated stature measurements. However, when repeated measurements are not available, studies often rely on predictive equations such as those proposed by Mirwald and colleagues [12] or Moore and colleagues [7], to assess timing of PHV.

Nevertheless, similar to Tanner staging, PHV provides information around PHV, and it is often categorised as pre-, circa, and post-PHV, limiting its utility in samples with adolescents well beyond PHV. Moreover, PHV estimates derived from Mirwald or Moore equations show increasing systematic prediction error with temporal distance from PHV [7, 8], with the largest errors occurring between ages of 15 and 17 years [13]. This corresponds closely to the age of participants at follow-up in our study [2], which was 15.8 ± 0.4 years. Most adolescents in our sample were also substantially post-PHV (~ 2.7 years (ranging from 0.8 to 4.6) from PHV) at the latest follow-up. In this context, the absence of direct sex hormone data and the post-PHV status of participants limit the usefulness of PHV-based indicators. Under these conditions, PHV-based maturation status would not be expected to outperform a clinician-based, sex-specific clinical pubertal staging. Because the utility of PHV as a maturational indicator decreases after the PHV-period, clinician-assessed pubertal staging may provide more accurate reflection of maturation in adolescents. Without direct sex hormone measurements, relying on PHV timing may fail to capture nuances in pubertal progression, especially in samples where participants are already beyond their peak growth phase. Sansum and colleagues [1] also noted that the strength of PHV is that the same metric can be used in girls and boys. However, in samples consisting of adolescents aged 15–17 years, the error is larger and utility smaller in girls than boys.

To conclude, only a limited number of studies have investigated associations between maturation and arterial stiffness or structure in adolescence. Evidence from longitudinal adult samples indicates that age at PHV, estimated using the SITAR model, does not predict arterial stiffness (PWV) or arterial structure (cIMT) at the age of 25 [14]. Similarly, in women, age at menarche has not been associated with cIMT assessed in adulthood, at ages 30–39 years [15]. To our knowledge, no studies have investigated how different indicators of maturation relate to arterial stiffness or structural measures in adolescents. This represents a notable gap in the literature, particularly given that adolescence is characterised by substantial hormonal, somatic, and cardiovascular changes. Future longitudinal research should therefore evaluate the feasibility and validity of alternative maturation assessments, including SITAR and PHV, clinician‑assessed secondary sex-characteristics, and hormone-based measures (e.g. urine or saliva) to identify the most appropriate approaches for minimising hormonal confounding and for isolating the independent effects of maturation on arterial stiffness and structure in addition to age, height and blood pressure in adolescence.

## Data Availability

Not applicable.

## Abbreviations

cIMT: Carotid intima–media thickness
PHV: Peak height velocity
PWV: Pulse wave velocity
SITAR: SuperImposition by Translation And Rotation
